# Feasibility and acceptability of a systematic offer of HIV rapid testing to Family Planning Centers visitors by non-physician professionals in France

**DOI:** 10.1371/journal.pone.0298507

**Published:** 2024-11-27

**Authors:** Pauline Penot, Julie Chateauneuf, Anne Simon, François Durand, Guillaume Barriere, Pierre Leroy, Nicolas Vignier

**Affiliations:** 1 Sexual Health Unit (CEGIDD), André Grégoire Hospital, GHT Grand Paris Nord Est, Montreuil, France; 2 Centre Population et Développement (UMR 196 Université Paris Descartes ‐ IRD), SageSud (ERL Inserm U 1244), Institut de Recherche pour le Développement, Paris, France; 3 André Grégoire Hospital, GHT Grand Paris Nord Est, Montreuil, France; 4 Department of Internal Medicine and Clinical Immunology, APHP, Hôpital Pitié-Salpêtrière, Sorbonne Université, University Pierre et Marie et Curie, Paris, France; 5 Medical Affairs, Gilead Sciences S.A.S., Boulogne-Billancourt, France; 6 Service de Maladies Infectieuses et Tropicales, Groupe Hospitalier Sud Ile-de-France, Melun, France; 7 Sorbonne Paris Nord University, UFR SMBH, IAME, INSERM UMR 1137, Department of Infectious and Tropical Diseases, Avicenne Hospital, University Hospitals Paris Seine-Saint-Denis, AP-HP, Bobigny, France; University of Zimbabwe Faculty of Medicine: University of Zimbabwe College of Health Sciences, ZIMBABWE

## Abstract

**Background:**

The Human Immunodeficiency Virus (HIV) epidemic remains active among women in Europe, with significant missed opportunities for HIV testing. Although HIV testing falls within the scope of Family Planning Centers (FPCs), it is often not offered. This pilot study assessed the feasibility and acceptability of systematically offering HIV rapid testing by non-physician professionals, independent of medical appointments, to visitors and accompanying persons in FCPs.

**Methods:**

In September 2020, three FPCs in the Paris region were selected for a 6-month pilot based on high local HIV diagnosis rates among women and the absence of an existing HIV rapid testing offer. A dedicated midwife coordinated a certified training course for non-physician professionals, including group discussions on obstacles and facilitators for offering HIV rapid testing, education on HIV and the use of HIV rapid tests, and the development of procedures, including a standardized HIV questionnaire, to systematically offer HIV rapid testing. We measured participation rates in the study and acceptability of the HIV rapid testing offer. We analyzed visitor characteristics, HIV risk factors and knowledge about HIV.

**Results:**

All non-physician professionals across the three FPCs were trained. HIV rapid testing was systematically offered to 261 incoming visitors and accompanying persons from September 2020 to March 2021: 93% completed the questionnaire, and 91% accepted the HIV rapid testing offer; 63% reported prior HIV risk behaviors. Visitors born in North Africa had the least knowledge about HIV. Twenty-two visitors declined the testing offer, citing recent HIV testing, no perceived lack of risk, feeling unprepared, or fear of the outcome.

**Conclusion:**

The results demonstrate the feasibility and strong acceptability of systematically offering HIV rapid testing by non-physician professionals in large urban FPCs. Extending this approach to include sexually transmitted infections (STIs) screening could be beneficial.

## Introduction

Human Immunodeficiency Virus (HIV) leads to Acquired Immunodeficiency Syndrome (AIDS) and remains a major public health concern, affecting over 2.3 million people in the World Health Organization (WHO) European Region as of 2022 [1]. The United Nations Program on HIV/AIDS (UNAIDS) has set the objective that 95% of people living with HIV (PLWH) wil. know their status by 2030 [2]. In France, it is estimated that there were 156,000 PLWH (0.2% of the population) in 2017, of whom 16% were unaware of their HIV status [3]. In 2022, around 17,000 new HIV infections were diagnosed in the European Union and European Economic Area (EU/EEA) [1], including between 4,200 to 5,700 in France, with 54% cases in heterosexual men and women, and 31% in women [1]. Furthermore, approximatively 30% of new HIV diagnoses in France occur at an advanced stage of the disease, despite the recommendations from the French National Authority for Health (Haute Autorité de Santé, HAS, 2017) for early HIV testing [4, 5], representing missed opportunities for early intervention. Late presenters cannot benefit from an early treatment to prevent possible secondary transmission and to reduce AIDS-related mortality [6].

Family planning encompasses various means of birth control, enabling families to decide whether to have children. The importance of family planning was recognized in the 1948 Universal Declaration of Human Rights and is uphelp by over 20 international conventions [7]. Family planning services may vary by country. In France, Family Planning Centers (FPCs) were established in 1974 following the legalization of contraception and were later renamed Sexual Health Centers in May 2022. They play a crucial role in promoting sexual and reproductive health, with a primary mission is to provide information, advice, and services related to contraception, sexuality, pregnancy, voluntary termination of pregnancy (VTP/abortion), and the prevention of sexually transmitted infections (STIs) [8]. These centers are designed to be accessible to everyone, including individuals under the age of 18 [5, 8], regardless of age, marital status, financial situation, or social background, with a commitment to confidentiality and anonymity [8].

In real life settings, the offer of HIV/STI testing in FPCs involves a complex and heterogeneous care pathway [9], with disparities between FPCs offering comprehensive care (including HIV, STIs, contraception and management of violence) and those where STI screening and treatment are limited [10]. Budget constraints are also cited as reasons for not systematically offering HIV/STI testing. In this context, educating staff on the importance of HIV/STI testing is essential to increase testing and prevention opportunities among visitors most at risk of HIV transmission, especially in those who do not perceive themselves to be at risk [2, 11].

Given this background, we initiated a pilot project to systematically offer HIV rapid testing in FPCs by non-physician professionals to all incoming visitors, including accompanying persons, independent of medical appointments. The non-physician professionals in FPCs were identified as key participants due to their interaction with all visitors, particularly in reception areas and waiting rooms. The study’s primary objective was to evaluate the feasibility of systematically offering HIV rapid testing in FPCs by non-physician professionals and measure the acceptability among incoming visitors and accompanying persons. Secondary objectives were to identify the obstacles encountered by the non-physician professionals and to profile the participants.

## Methods

### Study design and setting

This study was a pilot feasibility study conducted in three Family Planning Centers (FPCs) located in the in the Greater Paris Region: Intercommunal Hospital Center André-Grégoire (CHIAG) in Montreuil, Pitié-Salpêtrière Hospital (Public Healthcare-Paris Hospitals [AP-HP]) in Paris, and South Île-de-France Hospital Group (GHSIF) in Melun. The pilot was implemented from September 2020 to March 2021.

The participating FPCs were selected on the following cumulative criteria: 1) the FPC collaborated with hospitals but did not offer HIV rapid testing to any visitors or accompanying person, and the prescription of HIV serology during the medical consultations was very low; 2) The FPC was, an area with populations including people born abroad, young people, and those in precarious or vulnerable situations; 3) the FPC was in a high HIV diagnosis incidence area for women and heterosexual men, with an significant proportion of late HIV diagnoses, according to ANRS COINCIDE mapping [12].

The primary objective was to evaluate the feasibility and acceptability of systematically offering HIV rapid testing to all incoming visitors, including accompanying persons, by non-physician professionals, independently of any medical appointments. Feasibility was assessed by the participation rate of non-physician professionals in the FPCs, while acceptability was measured by the acceptance rate of the HIV testing offer among visitors and accompanying persons. The study’s secondary objectives were to identify the obstacles encountered by the non-physician professionals and to profile the participants.

### Study population

The study population comprised all visitors and their accompanying persons attending the three selected FPCs during the study period. Non-physician professionals working at these centers, including nurses, social and family counselors, nursing assistants, midwives, and administrative staff, also participated in the study, as they were responsible for offering and conducting the HIV rapid tests.

### Inclusion and exclusion criteria

All non-physician professionals and all incoming visitors and accompanying persons at the three selected FPCs were eligible to participate in the study. Inclusion criteria were defined as any non-professional physician working in the FPCs and any individual visiting the FPCs during the study period, regardless of their reason for the visit or their previous HIV testing history. Exclusion criteria included visitors who were unable or unwilling to provide consent.

### Training of non-physician professionals and procedures

A dedicated midwife was assigned to coordinate the study and ensure its implementation across the three FPCs. In September 2020, a two-day certified training course by CRISP Île-de-France was provided to all non-physician professionals in each FPC. This training included a 2-hour focus group discussion on obstacles and facilitators for offering and performing HIV rapid testing, up-to-date information on HIV, education on the use of HIV rapid test kits, and best practices, including how for communicating HIV testing outcomes. The midwife also coordinated the development of procedures and a standardized HIV questionnaire in the three FPCs to systematically offer HIV rapid testing to all incoming visitors.

Visitors and accompanying persons were asked about behaviors or situations that could have exposed them to HIV/STIs. After their initial responses, the non-physician professionals presented examples of such behaviors and situations and asked the visitors to reassess their exposure to HIV/STIs.

### Data collection

Data were collected from both non-physician professionals and FPC visitors.

Qualitative data regarding obstacles and facilitators to offering and performing HIV rapid testing were gathered during the 2-hour focus group discussions with non-physician professionals. The coordinating midwife attended the trainings as silent observer and collected the data anonymously in a diary. Quantitative data were collected anonymously from the visitors via a standardized HIV questionnaire (S1 Fig) administered face-to-face by the non-physician professionals and recorded in Sphinx software. The questionnaire captured demographic information, HIV risk factors, previous HIV testing history, and knowledge about HIV.

### Data analysis

The feasibility of the systematic offer of HIV rapid testing was assessed by observing the implementation process by the non-physician professionals across the three FPCs. Acceptability was measured by the proportion of visitors and accompanying persons who completed the HIV questionnaire and accepted the HIV testing offer. An interim review was planned after 100 HIV rapid tests were performed to evaluate the added value of the HIV questionnaire.

Visitor characteristics, including age, gender, place of birth, HIV testing history, risk factors, knowledge about HIV, and capacity to assess HIV infection risks were analyzed. Quantitative variables were expressed as numbers and percentages. Subgroup analyses were conducted to identify any patterns or correlations with decisions to decline the HIV rapid testing offer. Subgroups were compared using the chi-square test. Visitors’ self-assessment of their exposure to HIV/STIs before and after the presentation of examples was compared using the McNemar test.

The significance level was defined as p<0.05 for both statistical tests.

### Ethics, regulatory considerations, and data privacy

No ethical approval was required in compliance with the French Jardé law. The offer and performance of HIV rapid testing in adult individuals followed Article L1111-4 of the French Public Health Code [13] and the decree of May 28, 2010, published in the JORF n°0131 of June 9, 2010, which required only verbal consent (no documentation required). For individuals under the age of 18, the offer and performance of HIV rapid testing followed Article L.1111-5 and Article L.1111-5-1 of the French Public Health Code [14, 15]. At any time, visitors and accompanying persons were free to decline both the questionnaire and the HIV rapid testing offer. Visitors were informed of their rights under Article 13 of the French GDPR law, including the right to not participate and the right to access their responses. All visitors completing the questionnaire were given information regarding data collection and provided verbal informed consent. Data were collected anonymously, and a privacy impact assessment was conducted to ensure the compliance with the French and European General Data Protection Regulations (GDPR). The project was published in the online repository of the French Health Data Hub platform.

## Results

### Feasibility of the systematic offer of HIV rapid testing by non-physician professionals in Family Planning Centers

There was no prior HIV rapid testing offer in any of the three FPCs before the pilot. Eleven non-physician professionals were working in the FPCs (five nurses, two social and family counsellors, two nursing assistants, one midwife, and one secretary). All of them (11/11, 100%) were trained in September 2020. Finally, all of them implemented the HIV questionnaire face-to-face with visitors and systematically offered HIV rapid testing from September 2020 to March 2021.

#### Obstacles and facilitators to the systematic offer of HIV rapid testing by non-physician professionals

During the focus group discussions at the start of the trainings, non-physician professionals identified several obstacles to offering HIV rapid testing, including fear of delivering HIV-positive results, difficulties in discussing HIV/STIs and sexuality due to limited knowledge, and the perception that HIV/STI testing was outside the scope of FPCs, particularly given the availability of a dedicated STI clinic in the hospital. Additionally, HIV testing was sometimes perceived as an added workload. Examples of verbatim statements from non-physician professionals regarding these obstacles are presented in S3 Fig.

We categorized the responses in two main areas: 1) Obstacles related to non-physician professionals’ scientific knowledge and experience with HIV and its prevention, and facilitators to address these obstacles; 2) Obstacles related to the perception of the FPCs’ mission scope and facilities to perform HIV rapid testing, and facilitators to address these obstacles (Table 1).

**Table 1:** Obstacles and facilitators to the systematic offer of HIV rapid testing by non-physician professionals.

Category	Obstacles identified	Facilitators identified
**Lack of knowledge and experience with regard to HIV and its prevention**	• Feeling unqualified to administer a questionnaire on HIV risks and HIV rapid tresting • Difficulty discussing HIV/STIs and sexuality • Fear of announcing HIV-positive status	• Team training • Development of a HIV questionnaire and procedures for delivering HIV rapid test results • Strengthening of the link between STI clinics and point of contact physician who can be consulted in case of a positive result
**Perception of HIV/STI testing as not being within the scope of FPCs and facility limitations**	• Inconsistent prescription of STI/HIV serological testing by the medical teams • No budget allocated for free HIV rapid testing in the FPCs • HIV rapid testing perceived as an additional workload • Lack of dedicated place for HIV testing in FPC facilities	• Free provision of free HIV rapid tests as part of the pilot • Identification of a project coordinator and a point of contact in the FPC • Support during the face-to-face administration of the HIV questionnaire to visitors • Distribution of leaflets in the waiting room to facilitate the systematic offer of HIV rapid testing

FPC: Family Planning Center; HIV: Human Immunodeficiency Virus; STI: Sexually Transmitted Infection.

### Acceptability of systematically offering HIV rapid testing to visitors and accompanying persons

**Fig 1:**
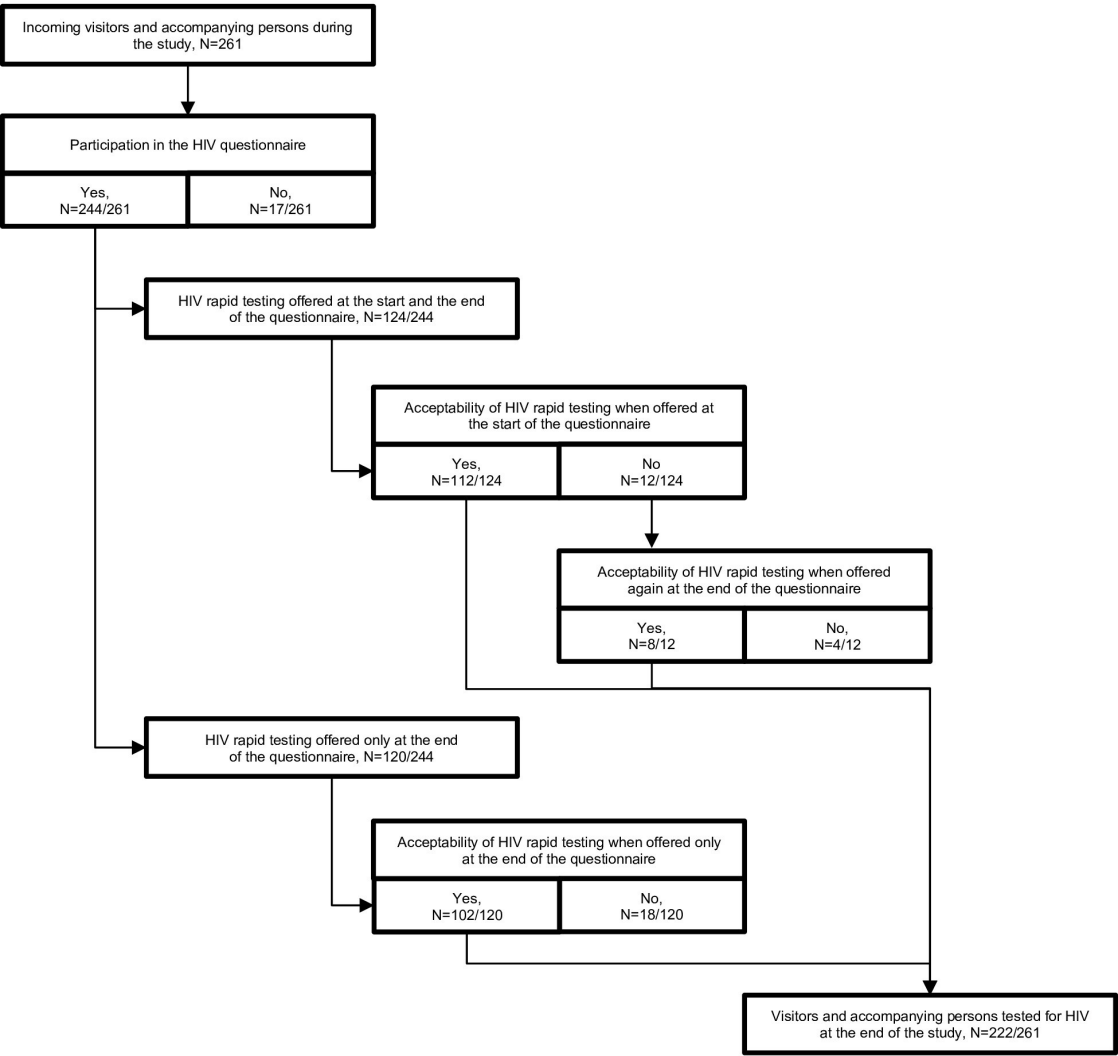
Participation in the study and acceptability of HIV rapid testing. HIV: Human Immunodeficiency Virus.

At the start of the pilot, the HIV rapid testing offer was at the beginning of the HIV questionnaire and, in case of refusal, offered again at the end. After 100 HIV rapid tests were carried out, the study team conducted an interim evaluation with the coordinating midwife to assess the added value of the HIV questionnaire. Among the 12 visitors who initially refused the HIV rapid test during this first part of the pilot, 8/12 (67%) changed their minds at the end of the HIV questionnaire and ultimately accepted the HIV rapid testing offer (Fig 1). It was concluded that the HIV questionnaire improved the acceptability of the HIV rapid testing offer and also served as an educational tool, enhancing visitor’s knowledge about HIV/STIs. Therefore, the study team decided that the HIV rapid testing systematic offer should be made only at the end of the HIV questionnaire. The coordinating midwife implemented this change across the three FPCs.

Finally, of the 261 visitors who entered one of the three FPCs during the pilot period from September 2020 to March 2021, 17 (7%) refused to participate in the study, while 244 (93%) completed the HIV questionnaire (Fig 1). Among the 244 visitors/accompanying persons who completed the HIV questionnaire, 222 (91%) accepted the HIV rapid testing offer (Fig 1). All HIV rapid test results were negative during the pilot period.

#### Characteristics of the visitors participating in the study

Among the 244 visitors who completed the questionnaire, 136 (56%) came in for a medical appointment, and 107 (44%) were accompanying persons. Of these, 166 (68%) were women; 142 (58%) were aged ≤ 25; 202 (83%) were of French nationality; 101 (40%) were born in West or North Africa; 122 (50%) were unemployed (61 (25%) were student and 61 (25%) were unemployed) (Table 2).

**Table 2:** Characteristics of the visitors participating in the study and their HIV testing history.

**Reason for visiting the FPC, n (%)**	
Medical appointment	136 (56)
Accompanying person	107 (44)
**FPC visited, n (%)**	
CHIAG	153 (63)
AP-HP Pitié Salpêtrière	54 (22)
GHSIF	36 (15)
Missing data	1 (0)
**Role of the FPC non-physician professional conducting the HIV questionnaire, n (%)**	
Midwife or midwife trainee	92 (38)
Social and family counsellor	71 (29)
Registered nurse	39 (16)
Nursing assistant	27 (11)
Secretary	15 (6)
**Sex, n (%)**	
Female	166 (68)
Male	78 (32)
**Age, n (%)**	
<18 years	29 (12)
18 to 25 years	113 (46)
26 to 35 years	58 (24)
>36 years	44 (18)
**Current administrative status, n (%)**	
French/European Union nationality	202 (83)
Temporary residence permit	28 (11)
Receipt of residence permit	3 (1)
Missing data	10 (4)
**Region of birth, n (%)**	
Africa	125 (49)
*West Africa*	*53 (21)*
*North Africa*	*48 (19)*
*Central Africa*	*18 (7)*
*East Africa*	*6 (2)*
Europe (WHO region)	81 (33)
*France*	*52 (21)*
*Outside of France*, *including Turkey*	*29 (12)*
Asia	16 (6)
French overseas territories	29 (12)
**Occupational status, n (%)**	
Permanent contract/Civil servant	85 (35)
Student/In training	61 (25)
Unemployed	61 (25)
Other	13 (6)
Temporary work	13 (5)
Fixed-term contract	10 (4)
Missing data	1 (0)
**Health insurance coverage, n (%)**	
Public basic with complementary insurance	138 (57)
Public basic without complementary insurance	45 (18)
Public basic with free public complementary insurance for low-income people	42 (17)
State medical assistance for undocumented migrants	7 (3)
None	12 (5)
**HIV testing history, n (%)**	
Yes	133 (54)
Last HIV testing date, n (%)	
*<3 months*	*10 (8)*
*Between 3 months and 1 year*	*25 (19)*
*>1 year*	*72 (54)*
*Non-respondent*	*26 (20)*
Reason for HIV testing, n (%)	
*Voluntary testing*	*104 (78)*
*Pregnancy monitoring/VTP*	*26 (20)*
*Voluntary testing and pregnancy monitoring/VTP*	*3 (2)*
No	99 (41)
Reason for not testing, n (%)	
(*Visitors were free to provide multiple answers)*	
*Not offered*	*60 (61)*
*I am not exposed to HIV*	*21 (21)*
*I fear the result*	*9 (9)*
*Test of my partner*	*7 (7)*
*I don’t feel ready*	*5 (5)*
*I don’t want to know*	*3 (3)*
*No answer*	*2 (2)*
I don’t know	12 (5)

AP-HP: Public Healthcare-Paris Hospitals (Paris); FPC: Family Planning and Education Center; CHIAG: Intercommunal Hospital Center André-Grégoire (Montreuil); GHSIF: South Île-de-France Hospital Group (Melun); WHO: World Health Organization; HIV: Human Immunodeficiency Virus; VTP: Voluntary Termination of Pregnancy

#### HIV testing history

Of the 244 visitor and accompanying persons who completed the HIV questionnaire, 99 (41%) had never been tested for HIV (Table 2). Among them, 60 (61%) reported that they had never been offered HIV testing, and 21 (21%) had not requested HIV testing yet because they did not consider themselves to be at risk of HIV. Subgroup analyses revealed that the proportion of visitors or accompanying persons who had never been tested for HIV was higher among those aged ≤ 25 compared to older individuals (52% vs. 29%; p = 0.0007). See S2 Fig with statistical contingency tables detailing significant subgroup analyses.

Of the 133 (54%) visitor and accompanying persons who had previously been tested for HIV, 104 (78%) had been tested voluntarily, while 20% had been tested during pregnancy monitoring or a voluntary termination of pregnancy.

#### Self-assessment of HIV/STI exposure

**Fig 2:**
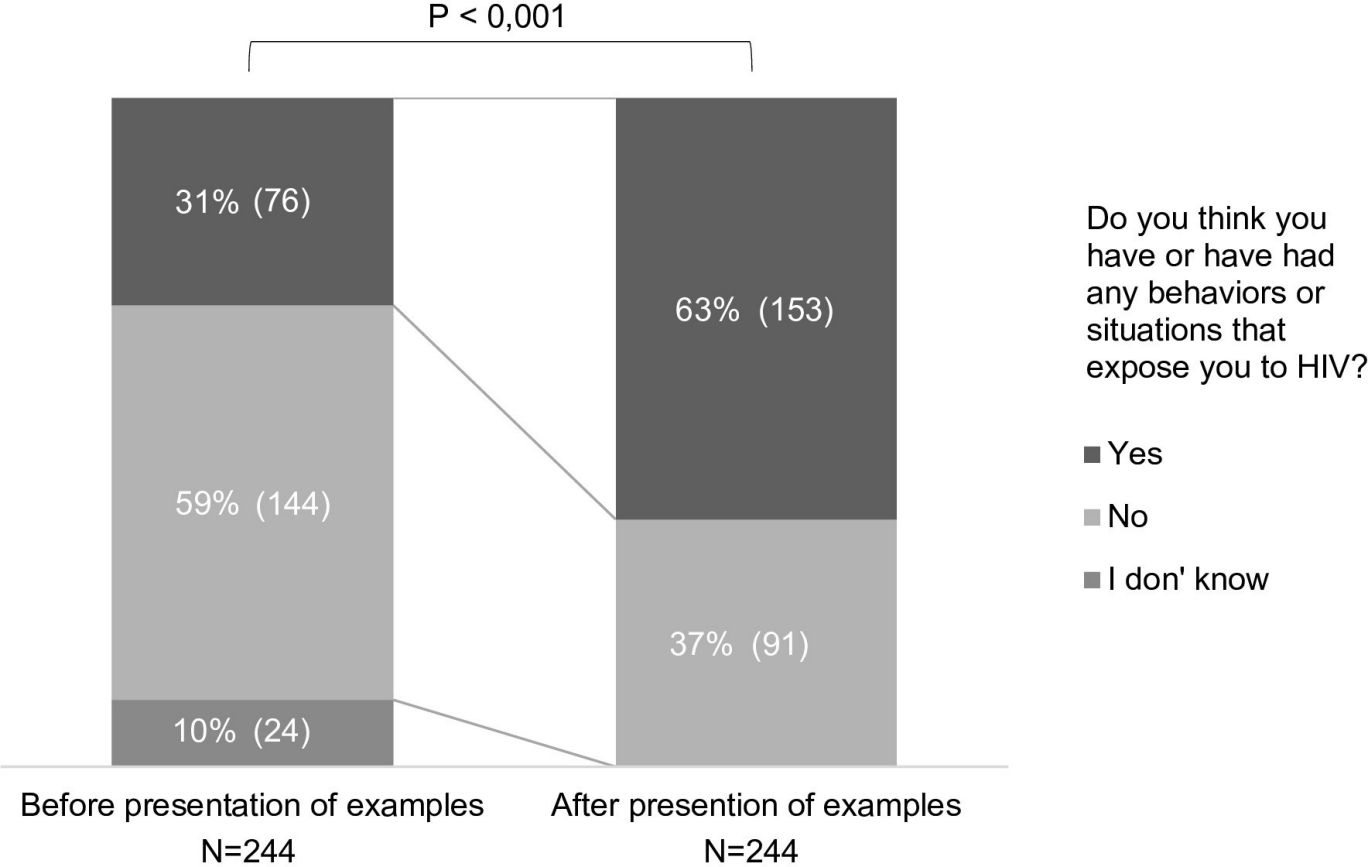
Self-assessment of HIV/STI exposure before and after presentation of examples. HIV: Human Immunodeficiency Virus; STI: Sexually Transmitted Infections.

Visitors and accompanying persons were asked about behaviors or situations that could have exposed them to HIV/STIs. After their initial responses, the non-physician professionals presented examples of such behaviors and situations and asked again the visitors to reassess their exposure to HIV/STIs. Initially, only 76 (31%) of visitors estimated that they had engaged in behaviors or situations that exposed them to HIV. After the presentation of examples, the proportion of visitor who estimated they had engaged in such behaviors or situations increased significantly to 153 (63%; p<0.05) (Fig 2).

Among those who, at the end of the questionnaire, estimated that they had engaged in behaviors or situations that exposed them to HIV, 109 (71%) were women, 93 (61%) were aged ≤25; 130 (85%) were of French or European Union nationality, 38 (25%) were born in West Africa, and 92 (60%) had visited the FPC for a medical appointment. Subgroup analyses indicated that the proportion of visitors or accompanying persons reporting such behaviors or situations was higher among individuals aged <18 compared to those in any of the three other age categories (79% vs. 62%, 67%, and 48%; p = 0.042) and was also higher among those reporting two or more sexual partners than among those reporting only one or no sexual partner (85% vs. 53%; p<0.001). See S2 Fig for statistical contingency tables detailing significant subgroup analyses.

#### Knowledge about HIV

Visitors and accompanying persons were asked about their knowledge about HIV (Table 3): 182 (75%) were unable to differentiate between HIV and AIDS terminologies; 161 (66%) did not know that PLWH receiving antiretroviral treatment and achieving viral suppression cannot transmit HIV, a concept known as U = U (Undetectable = Untransmissible), or were unaware of U = U; 134 answered these two questions incorrectly; 72 (30%) could not name a place where they could get HIV tested. Of the 134 visitors or accompanying persons who answered the two questions incorrectly: 93 (69%) were women; 61 (46%) were aged 18 to 25; 108 (81%) were of French or European Union nationality; 34 (25%) were born in North Africa; 23 (17%) were born in West Africa; and 134 (59%) had visited the FPC for a medical appointment. Subgroup analyses revealed that the proportion of visitors or accompanying persons who answered the two questions incorrectly was higher among individuals born in North Africa compared to those who were not (71% vs. 51%; p = 0.013). See S2 Fig with statistical contingency tables detailing significant subgroup analyses.

**Table 3:** Visitors’ knowledge about HIV.

**Difference between HIV and AIDS, n (%)**	
Yes	62 (25)
No	182 (75)
**Knowledge about U = U, n (%)**	
Yes	83 (34)
No	130 (53)
I do not know	31 (13)
**HIV testing sites spontaneously cited, n (%)**	
*(Visitors were free to provide multiple responses)*	
Family Planning Center	89 (36)
Laboratory	80 (33)
STI Screening Center	50 (20)
Healthcare center	39 (16)
Associations	20 (8)
Attending physician	15 (6)
Self-test	7 (3)
I don’t know	72 (30)

AIDS: Acquired Immunodeficiency Syndrome; HIV: Human Immunodeficiency; Virus; STI: Sexually Transmitted Infection. U = U: Undetectable = Untransmissible.

#### Characteristics of the visitors and accompanying persons who refused the HIV rapid testing offer

Of the 244 visitors or accompanying persons who completed the HIV questionnaire during the pilot, 22 (9%) refused the HIV rapid testing offer at the end. Their reasons for refusing were multiple and included recent HIV testing (11/22, 50%); perceived lack of risk (7/22, 32%); feeling unprepared (4/22, 18%); and fear of the results (3/22, 14%). Of those who refused, 16 (73%) were women; 15 (68%) were aged ≥26; 17 (77%) of French or European Union nationality; 7 (23%) born in West Africa. Subgroup analyses indicated that the proportion of visitors or accompanying persons refusing HIV testing was higher among those aged >25 than among younger individuals (15% vs. 5%; p = 0.009) and was higher among those reporting one or no sexual partner compared to those reporting more than two sexual partners (12% vs. 1% p = 0.008). See S2 Fig with statistical contingency tables detailing significant subgroup analyses.

## Discussion

Our results demonstrate the feasibility of systematically offering HIV rapid testing by FPC non-physician professionals, independently of a medical appointment, to all incoming visitors, including accompanying persons, with strong acceptability among those who might not have otherwise requested an HIV test.

In the FPCs, the non-physician professionals were identified as key because they interact with all the visitors and accompanying persons (e.g., at reception, in the waiting room). Therefore, all non-physician professionals, including administrative staff, nursing assistants, social and family counselors, and nurses, were recruited and trained for this pilot in the three participating FPCs. Physicians were not included because they don’t see all visitors or accompanying persons.

In our pilot, 41% of the incoming visitors or accompanying persons were tested for HIV for the first time. This rate was 10% higher compared to 2014 data from community testing reports using HIV rapid testing in France over a three-year period [16]. The main reason for having been tested previously was the lack of an HIV testing offer. This finding aligns with the literature [17] and highlights the need to promote HIV/STI testing in FPCs, especially among youth aged 25 or less. A two-year study conducted in the United States across 10 FPCs showed that systematically offering HIV testing to adolescents and young adults increased acceptability rates, test performance, and HIV diagnoses in this population [18]. This is particularly important in this pilot because those who had never been tested before were the youngest visitors, and adolescents represent the age group least likely to self-initiate HIV testing [19]. Knowledge about behaviors and situations exposing individuals to HIV was initially poor in the FPC population, and 21% of those who had never been HIV tested initially believed they were not at risk of HIV. The presentation of examples of such behaviors and situations during the HIV questionnaire led to a significant increase (32%) in visitors’ and accompanying persons’ ability to better assess their exposure to HIV, underscoring the need for HIV education in the general population.

This pilot also highlights the need for up-to-date HIV education among healthcare professionals. The focus group discussions during the training of the FCP non-physicians professionals identified a lack of knowledge and experience regarding HIV and its prevention as a major obstacle to offer HIV testing. The FCP non-physicians professionals are usually more knowledgeable about contraception and voluntary interruption of pregnancy, areas with which they feel more qualified [20]. This is particularly concerning since HIV testing is within the scope of FPCs [8]. The second major obstacle was the difficulty in discussing HIV and sexuality. Previous studies have also found this to be the case, especially with young people [21, 22]. The implementation of specific training and support in this pilot (e.g. appointing a project coordinator and a point of contact responsible for continuing the coordinator’s tasks; providing tools such as the HIV questionnaire; procedures for actions to be taken based on the HIV testing outcome; educational materials about HIV for visitors) likely helped overcome these obstacles, enabling the non-physician professionals to perform HIV rapid testing and communicate results with confidence and legitimacy. The literature also shows that, in many stings—from high to low-income countries (e.g. general practitioners [23], dental care, pharmacies, mobile units, emergency departments [24], migrant populations [25]…)—the main limitation of provider-initiated testing lies in the offer by health professionals, while the acceptability rate is high (i.e., people usually agree to get tested when offered) [25–27].

The HIV questionnaire revealed 75% of visitors did not know the difference between HIV and AIDS, and 66% were unaware of U = U (Undetectable = Untransmissible). Visitors and accompanying persons also had limited knowledge about where to get tested for HIV: 30% did not know any locations, and only 20% mentioned STI clinics as a testing site. The 2021–2024 French national sexual health strategy roadmap also identified the need to increase HIV awareness campaigns to improve access to sexual health and STI information globally, enabling people exposed to HIV to spontaneously request an HIV test and be linked to care [21].

This pilot had some limitations. Only three FPCs in the Greater Paris region were selected, and they may not be representative of the broader French and European landscape. However, these three centers were highly representative of large urban cities, with a high proportion of young visitors, many of whom were born outside the European Union and in precarious situations. In this pilot, we found most of the visitors were aged 25 or younger, were often born outside of France (especially in West and North Africa), and were in precarious situations. Due to budget constraints and the coordinating midwife’s workload, it was not possible to include more than three FPCs. This pilot also focused exclusively on HIV, whereas testing should ideally include other STIs, particularly screening for *Chlamydia trachomatis* among young people [28]. The qualitative data were collected by the coordinating midwife in a diary during the focus group discussions, where she acted as an auditor. The data collection was guided by the certified trainer’s methods. The 2-hour focus group discussions allowed to reach saturation in the responses from each FPC group as no new obstacles and facilitators emerged by the end. We did not conduct a formal qualitative analysis; instead, we reported all the obstacles and facilitators noted in the midwife’s diary.

This pilot was also challenged by the SARS-CoV-2 pandemic, as FPC and STI clinic staff focused on daily emergencies, leaving limited time to dedicate to HIV testing, which contributed to some inertia in its implementation in some centers.

Before this pilot, HIV rapid testing was not offered and not available in the three FPCs. The prescription of HIV serology by the physician team during the medical consultations was very low. When individuals were prescribed a HIV serology, they were referred to the local STI Clinic. Our results provide best practices for implementing a systematic offer of HIV rapid testing in FPCs, aligned with recommendations to extend FPC missions to include HIV and STI testing [29]. Expanding this pilot to all FPCs in France would be particularly valuable.

## Conclusion

This pilot demonstrated the feasibility of systematically offering HIV rapid testing by FPC non-physician professionals to incoming visitors and accompanying persons, even with limited resources, and showed strong acceptability of the HIV testing offer. Additionally, it highlighted the need for up-to-date education on HIV and the behaviors or situations that expose individuals to HIV/STIs for both FPC non-physician professionals and the visitors and accompanying persons.

These findings support the broader implementation of systematically offering HIV testing in FPCs to avoid missed opportunities for diagnoses and to enable comprehensive sexual health management. Extending this approach to include STIs screening could be beneficial.

## Supporting information

S1 Fig HIV standardized questionnaire. (DOCX)

S2 Fig Statistical contingency tables detailing significant subgroup analyses. (DOCX)

S3 Fig Examples of verbatim statements from non-physician professionals on obstacles to offering HIV rapid testing. (DOCX)
