# Supplementary material for: Feasibility and acceptability of a systematic offer of HIV rapid testing to Family Planning Centers visitors by non-physician professionals in France
Source: PLoS One. 2024 Nov 27;19(11):e0298507. doi: 10.1371/journal.pone.0298507 (PMC11602089; doi:10.1371/journal.pone.0298507)
Supplement: S1 Fig — (DOCX) [file pone.0298507.s001.docx]

**Figure S1. HIV standardized questionnaire.**

| **HIV QUESTIONNAIRE**  **Date**: [To be filled]  **Hospital**:   - CHIAG (Intercommunal Hospital Center André-Grégoire) - GHSIF (South Île-de-France Hospital Group) - AP-HP Pitié Salpêtrière (Public Healthcare-Paris Hospitals, Pitié-Salpêtrière Hospital)   **Structure**:   - FPC - STI clinic   **Position of the non-physician professional conducting the questionnaire:**   - Secretary - Registered nurse - Midwife or midwife trainee - Social and family counselor - Caregiver  1. **CHARACTERISTICS OF THE RESPONDENT**   **Type:**   - Visitor   What is the reason for your visit?   - Accompanying person   What is the reason for your visit?   - Other   **Sex:**   - Female - Male   **Age:** [To be filled]  **Current situation:**   - French/European Union nationality - Temporary residents permit - Receipt of residence permit - Undocumented   **Health insurance coverage:**   - State medical assistance for undocumented migrants - Public basic insurance with free public complementary insurance for low-income people - Public basic insurance with complementary insurance - Public basic insurance without complementary insurance - None   **Education level:**   - None - Primary school - Secondary school - College/University   **Occupational status:**   - Unemployed - Temporary work - Fixed-term contract - Student/ln Training - Permanent contract/Civil servant - Other   **Birthplace:** [To be filled]   1. **HIV RISK**   **Have you ever given birth in France?**   - Yes - No   **Have you ever had an abortion (VTP)?**   - Yes - No   **Have you ever had an HIV test?**   - Yes (voluntary screening) - Yes (pregnancy monitoring/VTP) - No   **If yes, when?**   - < 3months - Between 3 months to 1 year - > 1 year   **If no, reasons for refusal:** [To be filled]  *Do not list risk behaviors or situations until patient responds.*  **Do you think you have or have had any behaviors or situations that expose you to HIV?**   - Yes - No - I don't know   *List examples of behaviors or situations:*   - *Unprotected vaginal, oral, or anal intercourse.* - *Sharing unprotected sexual accessories or injection equipment?* - *Un know HIV status of partner(s)?* - *Sexual activity under the influence of drugs or alcohol?*   **After the presentation of these examples, do you think you have or have had any behaviors or situations that expose you to HIV?**   - Yes - No   **How many sexual partners have you had in the last 12 months?**   - None - 1 - 2 or more - I don't want to answer   **Have you recently had unprotected sex?**   - Yes - No   **If yes:**   - - < 72 hours   - 72 hours - 6 weeks   - 6 weeks - 3 months   - > 3 months  1. **KNOWLEDGE ABOUT HIV**   *(Do not list suggestions)*  **Do you know where you can get an HIV test?**   - Family Planning Center - Healthcare center - Associations - STI clinic - Laboratory - Other: - I don't know   **Do you know the difference between AIDS and HIV?**   - - Yes   - No   **In your opinion, can a person living with HIV receiving an effective antiretroviral treatment and achieving viral suppression transmit the virus during sexual intercourse?**   - - Yes   - No   - I don't know  1. **HIV RAPID TEST**   **Would you like to take an HIV rapid test now?**   - - Yes   - No   **If yes, outcome:**   - - - Positive     - Negative     - Unknown   **If no: why not?** [To be filled]  **At the end of the test,** what is the patient's destination?   - - Back home   - CPF   - STI Clinic   - Other   **Is an STI screening being carried out?**   - - Yes   - No   **Comments:** [To be filled] |
| --- |

AIDS: Acquired Immunodeficiency Syndrome; FPC: Family Planning Center; HIV: Human Immunodeficiency Virus; STI: Sexually Transmitted Infection; VTP: Voluntary Termination of Pregnancy.
