# Supplementary material for: Feasibility and acceptability of a systematic offer of HIV rapid testing to Family Planning Centers visitors by non-physician professionals in France
Source: PLoS One. 2024 Nov 27;19(11):e0298507. doi: 10.1371/journal.pone.0298507 (PMC11602089; doi:10.1371/journal.pone.0298507)
Supplement: S2 Fig — (DOCX) [file pone.0298507.s002.docx]

**Figure S2. Statistical contingency tables detailing significant subgroup analyses.**

**HIV testing history**

|  | | **Prior HIV-Testing** | |  |
| --- | --- | --- | --- | --- |
| **Age** (years) |  | No | Yes | Total |
| ⩽ 25 | N | 71 | 66 | 137 |
|  | % | 52% | 48% | 100% |
| ⩾ 26 | N | 28 | 67 | 95 |
|  | % | 29% | 70% | 100% |
| Total | N | 99 | 133 | 232 |
|  | % | 43% | 57% | 100% |

|  | value | df | p |
| --- | --- | --- | --- |
| 𝛘2 | 11.5 | 1 | 7.1 x 10-4 |
| N | 232 |  |  |

**Self-estimation regarding behaviors and situations exposing to HIV/STIs**

|  | | **Behaviors and situations**  **exposing to HIV/STIs** |  |
| --- | --- | --- | --- |
| **Age** (years) |  | Yes | Total |
| ⩽ 18 | N | 23 | 29 |
|  | % | 79% | 100% |
| 18 to 25 | N | 70 | 113 |
|  | % | 62% | 100% |
| 26 to 35 | N | 39 | 58 |
|  | % | 67% | 100% |
| ⩾ 36 | N | 21 | 44 |
|  | % | 47.7% | 100.0% |
| Total | N | 153 | 244 |
|  | % | 63% | 100% |

|  | value | df | p |
| --- | --- | --- | --- |
| 𝛘2 | 8.2 | 3 | 4.2 x 10-2 |
| N | 244 |  |  |

|  | | **Behaviors and situations**  **exposing to HIV/STIs** | |  |
| --- | --- | --- | --- | --- |
| **Number of sexual partners** | | No | Yes | Total |
| ⩽ 1 | N | 80 | 90 | 170 |
|  | % | 47% | 53% | 100% |
| ⩾ 2 | N | 11 | 61 | 72 |
|  | % | 15% | 85% | 100% |
| Total | N | 91 | 151 | 242 |
|  | % | 38% | 62% | 100% |

|  | value | df | p |
| --- | --- | --- | --- |
| 𝛘2 | 21.8 | 1 | 3.1 x 10-6 |
| N | 242 |  |  |

**Knowledge about HIV**

|  | | **2 wrong responses** | |  |
| --- | --- | --- | --- | --- |
| **Born in North Africa** |  | Yes | No | Total |
| Yes | N | 34 | 14 | 48 |
|  | % | 71% | 29% | 100% |
| No | N | 100 | 96 | 196 |
|  | % | 51% | 49% | 100% |
| Total | N | 134 | 110 | 244 |
|  | % | 55% | 45% | 100% |

|  | value | df | p |
| --- | --- | --- | --- |
| 𝛘2 | 6.1 | 1 | 1.3 x 10-2 |
| N | 244 |  |  |

**Acceptability of the HIV rapid testing offer by visitors and outcomes**

|  | | **Acceptability of the HIV rapid testing offer** | |  |
| --- | --- | --- | --- | --- |
| **Age (years)** |  | No | Yes | Total |
| < 25 | N | 7 | 135 | 142 |
|  | % | 5% | 95% | 100% |
| >26 | N | 15 | 87 | 102 |
|  | % | 15% | 85% | 100% |
| Total | N | 22 | 222 | 244 |
|  | % | 9% | 91% | 100% |

|  | value | df | p |
| --- | --- | --- | --- |
| 𝛘2 | 6.9 | 1 | 8.5 x 10-3 |
| N | 244 |  |  |

|  | | **Acceptability of the HIV rapid testing offer** | |  |
| --- | --- | --- | --- | --- |
| **Number of sexual partners** | | No | Yes | Total |
| ⩽ 1 | N | 20 | 150 | 170 |
|  | % | 12% | 88% | 100% |
| ⩾ 2 | N | 1 | 71 | 72 |
|  | % | 1% | 99% | 100% |
| Total | N | 21 | 221 | 242 |
|  | % | 9% | 91% | 100% |

|  | value | df | p |
| --- | --- | --- | --- |
| 𝛘2 | 6.9 | 1 | 8.8 x 10-3 |
| N | 242 |  |  |

HIV: Human Immunodeficiency Virus; STIs: Sexually Transmitted Infections.
