# Supplementary material for: Feasibility and acceptability of a systematic offer of HIV rapid testing to Family Planning Centers visitors by non-physician professionals in France
Source: PLoS One. 2024 Nov 27;19(11):e0298507. doi: 10.1371/journal.pone.0298507 (PMC11602089; doi:10.1371/journal.pone.0298507)
Supplement: S3 Fig — (DOCX) [file pone.0298507.s003.docx]

**Figure S3. Examples of verbatim statements from non-physician professionals on obstacles to offering HIV rapid testing.**

|  | **Examples of verbatim statements from non-physician professionals on obstacles to offering HIV rapid testing** |
| --- | --- |
| **Lack of knowledge and experience related to HIV and its prevention** | |
| Feeling of inadequate of qualification to conduct an HIV risks questionnaire and perform HIV rapid testing | *"I'm a social worker. I don't feel I have the right to offer an HIV test, I haven't received any training."*  *"If people ask me questions during the questionnaire, I'm not sure if I'll be able to answer them."* |
| Difficulty discussing HIV/STIs and sexuality | *"I'm a bit hesitant about asking certain questions regarding the sexual lives of the people I'll have to interview"* |
| Fear of disclosing HIV status | *"I'm afraid to announce a positive test result"* |
| **Perception of HIV/STI testing as outside the scope of FPC services and facility limitations** | |
| Inconsistent prescription for HIV/STI serological testing by the medical teams | *"Some doctors don't prescribe STI or HIV tests because they don't have the time to focus on the main request of the consultation."* |
| No budget allocated for the cost of free HIV rapid testing in the FPC | *"We don't have the budget to perform a HIV Rapid Test on all the people who enter the center."* |
| HIV rapid testing perceived as an additional workload | *"We've already got a lot of projects ongoing, so it's difficult for us to get involved in another project at the moment."*  *"I thought the HIV test would be done quickly and*  *I hadn't considered the time needed for the HIV questionnaire."*  *"STI and HIV testing is not part of our mission.*  *I see it as an additional task, more work."* |
| No dedicated space for HIV testing in FPC facilities | *"I don't have space to perform the HIV Rapid Test in the office"*  *"There is no dedicated room at the moment to perform the test."* |

FPC: Family Planning Center; HIV: Human Immunodeficiency Virus; STI: Sexually Transmitted Infection.
